# Significance of cardiovascular comorbidity in patients with chronic myelomonocytic leukemia

**DOI:** 10.1007/s10354-022-00982-7

**Published:** 2022-11-21

**Authors:** David Lackner, Klaus Geissler

**Affiliations:** 1 Medical School, Sigmund Freud University, Vienna, Austria; 2 Department of Internal Medicine V with Hematology, Oncology and Palliative Care, Hospital Hietzing, Wolkersbergenstr. 1, 1130 Vienna, Austria

**Keywords:** Chronic myelomonocytic leukemia, Cardiovascular disease, Survival, Time to transformation, Coronary heart disease, Chronische myelomonozytäre Leukämie, Kardiovaskuläre Erkrankungen, Überleben, Dauer bis zur Transformation, Koronare Herzkrankheit

## Abstract

In a retrospective study, we analyzed the prevalence of common cardiovascular comorbidities in 310 patients with chronic myelomonocytic leukemia (CMML), their potential prognostic impact, and potential correlations with laboratory and molecular features. 115 (36%) patients had a documented cardiovascular comorbidity. In these patients, coronary heart disease 41/115 (36%), atrial fibrillation 34/115 (29%), and hypertension 75/115 (64%) were documented. None of these conditions had a significant impact on survival. Unexpectedly, patients with cardiovascular comorbidity had a lower number of circulating blasts and a lower prevalence of *EZH2* mutations. Moreover, time to transformation was significantly longer in these patients. Cardiovascular comorbidity does not seem to have a major impact on prognosis in CMML patients. The unexpected lower transformation rate in these patients needs to be further investigated.

## Abstract (de)

In einer retrospektiven Studie wurde die Prävalenz häufiger kardiovaskulärer Komorbiditäten bei 310 Patienten mit chronischer myelomonozytärer Leukämie (CMML) analysiert, ihr potenzieller prognostischer Einfluss sowie mögliche Korrelationen mit laborchemischen und molekularen Eigenschaften. Bei 115/310 (36 %) Patienten bestand mindestens eine dokumentierte kardiovaskuläre Komorbidität. Unter diesen Patienten wiesen 41/115 (36 %) eine koronare Herzkrankheit, 34/115 (29 %) Vorhofflimmern und 75/115 (64 %) einer arterielle Hypertonie auf. Keine dieser Komorbiditäten hatte einen signifikanten Einfluss auf das Überleben. Unerwarteterweise hatten Patienten mit kardiovaskulärer Komorbidität eine geringere Anzahl zirkulierender Blasten und eine geringere Prävalenz von *EZH2*-Mutationen. Darüber hinaus war bei diesen Patienten die Zeitdauer bis zur Transformation länger. Kardiovaskuläre Komorbiditäten scheinen keinen wesentlichen Einfluss auf die Prognose bei CMML-Patienten zu haben. Die unerwartete geringere Transformationsrate bei Patienten mit kardiovaskulären Komorbiditäten bedarf weiterer Untersuchungen.

## Introduction

Chronic myelomonocytic leukemia (CMML) is a rare, genotypically and phenotypically heterogenous hematologic malignancy of elderly people with an intrinsic risk to progress and transform into secondary acute myeloid leukemia (AML). With regard to the presence of myeloproliferation, CMML was originally subdivided into myeloproliferative disorder (MP-CMML; white blood cell [WBC] count >13 × 10^9^/L) versus myelodysplastic syndrome (MD-CMML; WBC count ≤13 × 10^9^/L) by the FAB criteria [1, 2]. Since CMML is characterized by features of both MDS and MPN, the World Health Organization (WHO) classification of 2002 assigned CMML to the mixed category, MDS/MPN [3]. CMML is further subclassified by the WHO into three groups based on blast equivalents (blasts plus promonocytes) in peripheral blood (PB) and bone marrow (BM) as follows: CMML‑0 if PB < 2% and BM < 5% blast equivalents; CMML‑1 if PB 2–4% or BM 5–9% blast equivalents; and CMML‑2 if PB 5–19% or BM 10–19% blast equivalents, and/or Auer rods are present [4]. CMML patients may have a highly variable outcome, suggesting that several factors can determine the course of disease and the cause of death in these patients [5–9]. There are a number of established prognostic parameters that have been incorporated into several prognostic models [10–21].

Cardiovascular disease is the leading cause of death in the general population [22]. Since CMML is a disease of elderly patients, cardiovascular disease may significantly impact the survival of these patients. The clinical significance of cardiovascular comorbidity in CMML is poorly investigated. Using the database of the Austrian Biodatabase for Chronic Myelomonocytic Leukemia (ABCMML), we analyzed 310 CMML patients with available information on cardiovascular comorbidity [23]. These information from our real-life database could be useful in the management of patients with CMML.

## Patients and methods

### Patients

Recently, we have shown that the ABCMML may be used as a representative and useful real-life data source for biomedical research [23]. In this database, we retrospectively collected epidemiologic, hematologic, biochemical, clinical, immunophenotypic, cytogenetic, molecular, and biologic data of patients with CMML from different centers. The diagnosis of CMML and leukemic transformation was according to the WHO criteria [2–4]. Cardiovascular comorbidities were defined according to criteria that were considered standard at the time of CMML diagnosis. Clinical and laboratory routine parameters were obtained from patient records. A detailed central manual retrospective chart review was carried out to ensure data quality before analysis of data from institutions. Due to the fact that CMML may be considered as an evolutionary process from clonal hematopoiesis of indeterminate potential (CHIP) to CMML-related AML [24], and the fact that the distinction between mature and immature monocytic cells, which is required to determine the time of transformation into AML, is notoriously difficult due to the lack of reliable immunophenotypic markers, we found it more appropriate not to exclude the CMML patients with transformation from our analysis [25].

In 310 CMML patients collected between 01.01.1990 and 31.03.2019, information was available regarding cardiovascular comorbidity. This research was approved by the ethics committee of the City of Vienna on 10 June 2015 (ethics code: 15-059-VK).

### Molecular studies

Genomic DNA was isolated from mononuclear cell (MNC) fractions of the blood samples according to standard procedures. The mutational status of CMML-related protein-coding genes was determined by targeted amplicon sequencing using the MiSeq platform (Illumina, San Diego, CA, USA). Details regarding gene panel, library preparation, and data processing have been reported previously [23]. Only variants with an allelic frequency (VAF) ≥ 5%, a described population frequency (MAF) < 1%, and an annotated pathogenic effect (or probability > 90% of being pathogenic) were included, with pathogenicity determined according to public databases and published studies.

### Statistical analysis

The log-rank test was used to determine whether individual parameters were associated with overall survival (OS). OS was defined as the time from sampling to death (uncensored) or last follow-up (censored). Time to transformation was defined as the time from sampling to transformation into AML (uncensored) or last follow-up (censored). Dichotomous variables were compared between different groups with the chi-square test. The Mann–Whitney U test was used to compare two unmatched groups when continuous variables were nonnormally distributed. Results were considered significant at *p* < 0.05. Statistical analyses were performed with SPSS v. 27 (IBM Corp., Armonk, NY, USA); the reported *p*-values were two-sided.

## Results

### Patient characteristics

The baseline characteristics of the 310 patients with CMML are shown in Table 1. In order to make comparisons with other published CMML cohorts possible, the percentages of patients regarding established prognostic parameters are given. As seen in other CMML series, there was a male predominance among study patients and more than half of the patients were aged 70 years or older [17]. The proportion of patients with leukocytosis > 13 G/L, thrombocytopenia < 100 G/L, and the presence of blast cells in peripheral blood was higher as compared to other series, indicating that our cohort included a higher number of patients with more advanced disease [17]. Indeed, 31 patients in this cohort had already transformed into CMML-related acute myeloid leukemia (AML) at time of study inclusion.

**Table 1:** Characteristics of CMML patients

	Cases *N* = 310	Percent (%)
*Age* *Evaluable* *=* *310*		
< 70 years	112	36
≥ 70 years	201	64
*Sex* *Evaluable* *=* *310*		
Male	190	61
Female	120	39
*Leukocytes* *Evaluable* *=* *308*		
> 13 G/L	150	49
≤ 13 G/L	158	51
*Hemoglobin* *Evaluable* *=* *308*		
< 10 g/dL	108	35
≥ 10 g/dL	200	65
*Platelets* *Evaluable* *=* *308*		
< 100 G/L	149	48
≥ 100 G/L	159	52
*Peripheral blood blasts* *Evaluable* *=* *275*		
Present	129	47
Absent	146	53

*CMML* chronic myelomonocytic leukemia

### Prevalence of cardiovascular comorbidity in CMML

A total of 115/310 (37%) patients had a documented cardiovascular comorbidity. In these patients, coronary heart disease 41/115 (36%), atrial fibrillation 34/115 (29%), and hypertension 75/115 (64%) were documented.

### Impact of cardiovascular comorbidity on clinical outcome

**Fig. 1:**
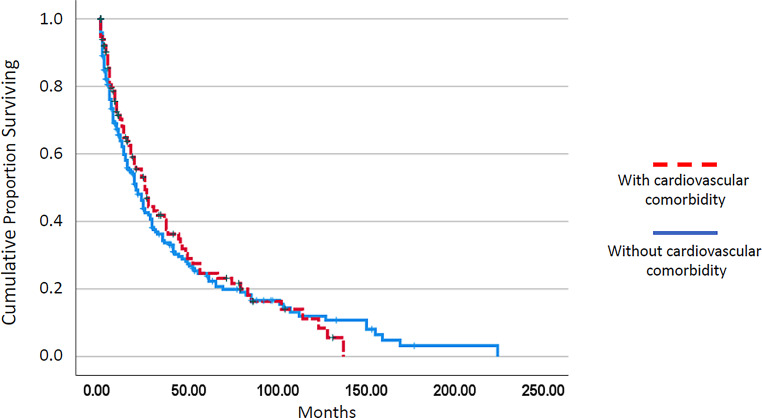
Kaplan–Meier plots for overall survival in chronic myelomonocytic leukemia patients with and without cardiovascular comorbidity

**Fig. 2:**
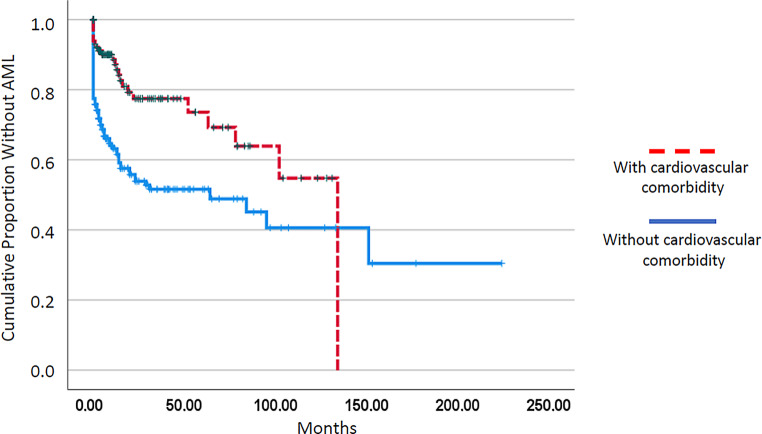
Kaplan–Meier plots for time to transformation in chronic myelomonocytic leukemia patients with and without cardiovascular comorbidity

As shown in Fig. 1, the median survival of patients with cardiovascular comorbidity was not significantly different from that in patients without cardiovascular comorbidity (25 vs. 20 months, *p* = 0.638). Among established prognostic parameters including leukocytosis > 13 G/L, anemia < 10 g/dL, thrombocytopenia < 100 G/L, and the presence of blast cells in peripheral blood, all of them had a highly significant adverse impact on survival in the univariate analysis (Table 2). The univariate analyses for coronary heart disease, atrial fibrillation, and hypertension are given in Table 3. There was no impact on survival in any of these comorbidities. Interestingly, in patients with cardiovascular comorbidity, time to transformation into CMML-related AML was significantly longer (Fig. 2; median time to transformation 134 vs. 64 months, *p* = 0.000).

**Table 2:** Univariate analysis of established single prognostic parameters in patients with CMML

Factors	Factor present Median OS (months)	Factor absent Median OS (months)	*P*-value (Log-rank)
WBC > 13 × G/L	17.0	29.0	0.000
Hb < 10 g/dL	11.0	28.0	0.000
PLT < 100 × G/L	13.0	30.0	0.000
PB blasts present	13.0	35.0	0.000

The log-rank test was used to determine if individual parameters were associated with OS

*CMML* chronic myelomonocytic leukemia, *OS* overall survival, *WBC* white blood cell count, *Hb* hemoglobin, *PLT* platelet count *PB* peripheral blood

**Table 3:** Univariate analysis of cardiovascular comorbidities in patients with CMML

Comorbidity	Comorbidity present Median OS (months)	Comorbidity absent Median OS (months)	*P*-value (Log-rank)
Coronary heart disease	26.0	21.0	0.519
Atrial fibrillation	25.0	23.0	0.955
Hypertension	27.0	20.0	0.388

The log-rank test was used to determine if individual parameters were associated with OS

*CMML* chronic myelomonocytic leukemia, *OS* overall survival

### Correlation of cardiovascular comorbidity with laboratory and molecular features

As shown in Table 4, the proportion of males among CMML patients with cardiovascular comorbidities was higher as compared to patients without these comorbidities. There was no difference in CMML patients stratified by the presence or absence of cardiovascular comorbidity regarding leukocyte counts, hemoglobin values, and platelet counts. However, the number of circulating blasts was lower in CMML patients with cardiovascular comorbidity. Regarding molecular aberrations, patients with cardiovascular comorbidity had a lower prevalence of *EZH2* mutations (Table 5). Furthermore, there was a trend toward a lower proportion of mutations in *SETBP1* and in these patients. Moreover, there was a trend toward a higher proportion of *TET2* mutations in CMML patients with cardiovascular comorbidity.

**Table 4:** Laboratory features stratified by the presence or absence of cardiovascular comorbidity

	All patients (*N* = 310)	With cardiovascular comorbidity (*n* = 115)	Without cardiovascular comorbidity (*n* = 195)	*P*-value
Age in years; median (range) Evaluable = 310	73 (34–92)	73 (52–92)	72 (34–92)	0.469
Sex (male); *n* Evaluable = 310	190 (61%)	88 (77%)	102 (52%)	0.000
Leukocytes G/L; median (range) Evaluable = 308	12.6 (0.7–238)	12.8 (2.6–140)	12.5 (0.7–238)	0.792
Hemoglobin g/dL; median (range) Evaluable = 308	10.9 (5.1–16.7)	10.9 (6.1–16.5)	10.9 (5.1–16.7)	0.874
Platelets G/L; median (range) Evaluable = 308	104 (2–709)	109 (5–695)	99 (2–709)	0.838
PB blasts %; median (range) Evaluable = 275	0 (0–87)	0 (0–69)	1 (0–87)	0.001

**Table 5:** Molecular features stratified by the presence or absence of cardiovascular comorbidity

Mutated gene VAF (≥ 5%)	With cardiovascular comorbidity	Without cardiovascular comorbidity	*P*-value
*NRAS*	14/65 (22%)	11/55 (20%)	0.836
*KRAS*	8/65 (12%)	7/55 (13%)	0.945
*CBL*	9/65 (14%)	10/55 (18%)	0.517
*NF1*	4/55 (7%)	6/47 (13%)	0.352
*PTPN11*	3/65 (5%)	5/55 (9%)	0.327
*SETBP1*	14/65 (22%)	20/55 (36%)	0.073
*JAK2*	12/65 (18%)	7/55 (13%)	0.391
*TET2*	51/65 (78%)	36/55 (65%)	0.112
*IDH1/2*	4/65 (7%)	5/54 (7%)	0.524
*ASXL1*	14/65 (22%)	16/55 (29%)	0.341
*EZH2*	6/65 (9%)	16/55 (29%)	0.005
*DNMT3A*	5/65 (8%)	7/55 (13%)	0.360
*SRSF2*	19/65 (29%)	15/55 (27%)	0.813
*ZRSR2*	5/65 (8%)	5/55 (9%)	0.782
*U2AF1*	9/65 (14%)	8/55 (15%)	0.913
*SF3B1*	2/64 (3%)	6/55 (11%)	0.091
*RUNX1*	7/65 (11%)	10/55 (18%)	0.246
*TP53*	13/64 (20%)	11/54 (20%)	0.994

*VAF* variant allele frequency

## Discussion

Cardiovascular morbidity is still the most common cause of mortality in people from European countries [22]. Since patients with CMML are often elderly and death in this cohort may be due to leukemia-related causes but also from non-leukemia-related causes, it is of interest to analyze whether cardiovascular comorbidity has an impact on survival in these patients. Our data show that this is obviously not the case, since the survival of patients with cardiovascular comorbidity was not different from patients with cardiovascular comorbidity. This was seen in all common subgroups of cardiovascular comorbidity such as coronary heart disease, atrial fibrillation, and hypertension. This finding is new and has, to the best of our knowledge, not been reported before.

Regarding other factors that may impact survival, the study cohort was comparable with CMML series reported by others. Among these established single prognostic parameters are leukocytosis > 13 G/L, anemia < 10 g/dL, thrombocytopenia < 100 G/L, and the presence of blast cells in peripheral blood. All these factors had a highly significant adverse impact on survival, indicating that the patient cohort we used in this study was comparable with CMML patient series published by others.

By comparing laboratory and molecular features between CMML patients with or without cardiovascular comorbidity, however, we found a lower number of circulating blasts in patients with as compared to patients without cardiovascular comorbidity. We also found in the molecular analysis a lower prevalence of *EZH2* mutations in patients with cardiovascular comorbidity. Finally, time to transformation was significantly longer in these patients. We and others have shown that circulating blasts are an established adverse prognostic factor in patients with CMML [6, 23]. We have recently demonstrated that a composite molecular parameter including *NRAS/CBL/EZH2*, derived from its impact on spontaneous in vitro myeloid colony formation, was predictive for inferior survival as well as for an increased risk of transformation [26]. In the multivariable analysis reported by the Mayo group for their prognostic model, PB blasts but not BM blasts had a significant impact on leukemia-free survival [6]. This may provide an explanation for the shorter time to transformation which was observed in patients with cardiovascular comorbidity. Altogether, these data suggest that in our study, patients with cardiovascular comorbidity had less advanced CMML.

This finding is unexpected and provocative, and certainly needs to be explored by further studies. At the moment, it is unclear what this observation could mean and is just a matter of speculation. Due to the population-based nature of our study, we cannot exclude a selection bias favoring referral of patients with less comorbidity but more clinically aggressive disease by physicians in the community. In our study, a proportion of CMML patients was not primarily seen by an experienced center and therefore there is a possibility that patients with advanced CMML but not considered for treatment by their community physician because of severe comorbidities were not sent to a center of competence, resulting in a shift towards a higher proportion of more favorable patients in the cohort of patients with cardiovascular comorbidity. A second explanation could be more regular and better management of these CMML patients because of their comorbidities. Finally, a third and most exciting explanation could be that the medication which is taken by the patient for cardiovascular problems may have some unknown beneficial impact on the progression of CMML. Considering the role of inflammation in the progression of myeloid disorders, a higher consumption of anti-inflammatory drugs, particularly of acetylsalicylic acid, could mitigate chronic inflammation in these patients [27, 28]. Based on the available data in our dataset, it is not possible to prove or disprove this hypothesis. Therefore, these findings, which clearly need further attention, are hypothesis generating at best, and need to be validated by others before they are used as a basis for new potential treatment concepts.

There is some evidence in the literature supporting a link between *TET2* mutations and cardiovascular disease. In 2014, independent epidemiological studies revealed that CHIP was associated with a substantial increase in the risk of all-cause mortality [29]. Unexpectedly, it was revealed that this increase in all-cause mortality could, at least in part, be attributed to a large increase in the frequency and death due to atherosclerotic cardiovascular conditions, such as coronary heart disease and ischemic stroke [30]. Specifically, it has been found that hematopoietic mutations in common driver genes, *DNMT3A, TET2*, and *JAK2*^*V671F*^, can accelerate experimental atherosclerosis and/or heart failure by generating a pool of myeloid cells with an augmented proinflammatory profile [31]. In our study, we also looked for a potential difference in the proportion of mutations between patients with or without cardiovascular comorbidity. In our study, there was a trend toward a higher proportion of *TET2* mutations in CMML patients with cardiovascular comorbidity.

We are aware of the limitations of our study. For example, most of the information used in this study was derived from retrospective real-world data that were not collected systematically or prospectively. Therefore, significant confounding by unknown parameters cannot be excluded, as discussed above. Moreover, not every parameter was available in all patients: data from patient records were obtained over many years and from many different centers, and the patients included in this study were a relatively heterogenous population regarding the blast cell counts. However, real-world data have recently been recognized as an important way to get insights into the routine management and natural history of rare diseases [32]. CMML is a rare disease and adequate patient numbers for a systematic and prospective study are not easy to collect within a limited timeframe. Moreover, the ABCMML provides information derived from molecular as well as from functional studies and therefore allows a more comprehensive view and deeper insight into the complex pathophysiology of this hematologic malignancy [23].
